# Effects of Basic Psychological Needs on Resilience: A Human Agency Model

**DOI:** 10.3389/fpsyg.2021.700035

**Published:** 2021-08-31

**Authors:** Yuan Liu, Xiaoxing Huang

**Affiliations:** ^1^Faculty of Psychology, Southwest University, Chongqing, China; ^2^Beijing Key Laboratory of Applied Experimental Psychology, School of Psychology, Beijing Normal University, Beijing, China; ^3^The People's Hospital of Guangxi Zhuang Autonomous Region, Guangxi, China

**Keywords:** academic resilience, relatedness, competence, autonomy, academic performance

## Abstract

Academic resilience refers to the ability to recover and achieve high academic outcomes despite environmental adversity in the academic setting. At the same time, self-determination theory (SDT) offers a human agency model to understand individuals' autonomy to achieve in various fields. The present longitudinal study explored the factors influencing resilience from the analytical framework of SDT to investigate how basic psychological needs strengthen students' resilience. A mediation model was proposed that resilience may mediate the relationship between basic psychological needs and academic performance. The results from 450 10th grade Chinese students showed that three basic psychological needs (i.e., autonomy, competence, and relatedness) facilitate academic resilience; academic resilience thus increases subsequent academic performance after controlling for previous test scores.

## Introduction

### Resilience Concept and Its Influence in Educational Field

Resilience is a broad psychological construct, which, the study of resilient students has gone through its very first stage focused its definition from the 1970s to the end of the last century (Wright et al., 2013). Terminology of resilience, such as risk, adversity, protective factor, recovery, sustain, and other relevant keywords are explored (Masten et al., 1990; Werner and Smith, 1992; Masten, 1994). Until now, the analytical scope of resilience not only focused on its connotation and denotation, but it also expanded to the self-reference or self-regulation processes such as self-esteem, self-confidence, and self-transcendence (Phillips-Salimi et al., 2007).

In the field of education, academic resilience is defined as after protective behavior when students meet the background adversities but achieve high academic outcomes (OECD, 2021). This means that students still get a high achievement even when they are confronted with the setback of fewer home educational resources (Cappella and Weinstein, 2001; Youssef and Luthans, 2007; Yun et al., 2018; Aydin and Michou, 2019; Sattler and Gershoff, 2019). Sometimes, “academic buoyancy” is regarded as a relevant concept in the schooling lives of students, referring to how students overcome or “bounce back” from everyday academic adversity (Martin and Marsh, 2008). In the Organization for Economic Cooperation and Development (OECD), a student is classified as resilient if he or she is in the bottom quarter of the index of economic, social, and cultural status in the country/economy of assessment and performs in the top quarter of students among all countries/economies, after accounting for socioeconomic status (OECD, 2003, 2013, 2016).

Particularly, recent studies indicate that mathematical skills are related to individuals' self-regulation. Not only do the self-regulation skills of children, including learning persistence, support their future math learning even in adulthood, but also early math experiences of children predict growth in their self-regulation process (McClelland et al., 2013; Schmitt et al., 2017; DeFlorio et al., 2019). Meanwhile, the domain of mathematics has larger gains and losses in school compared with reading since the home environments of all the children tend to provide more out-of-school opportunities to practice reading than mathematics skills (Cooper et al., 1996). This shows that self-reference processes of students play a prominent role in the domain of mathematics (Borman and Overman, 2004; Sandoval-Hernández and Białowolski, 2016).

### Basic Psychological Needs as Predictors of Resilience

The basic psychological needs theory (BPNT; Deci and Ryan, 2002) is one of the mini theories in self-determination theory (Deci and Ryan, 1985, 2000; Ryan and Deci, 2000, 2020; SDT) that emphasizes the intrinsic motivation and addresses three basic needs of human beings that are inherent as inner motivational resources. Basic psychological needs are needs for autonomy, competence, and relatedness (Ryan and Deci, 2000, 2017). The three psychological needs indeed play a prominent role in development, adjustment, and wellness across cultures, with strong implications for basic motivational domains, applied practices, and even broad social policies (Gillison et al., 2019).

The basic psychological needs indeed may improve the level of resilience. For example, in two longitudinal programs, the Kauai longitudinal study and the Minnesota parent-child project, they have shown that individuals who availed themselves of informal sources of support in the community, and whose lives subsequently took a positive turn, differed in significant ways from those who did not make use of such options (Werner and Smith, 2001; Yates et al., 2003; Werner, 2013). Meanwhile, scholastic competence at age 10 was also positively linked to self-efficacy and the ability to make realistic plans at age 18 (Werner and Smith, 2001; Yates et al., 2003; Werner, 2013). Moreover, boys who were more autonomous at age 2 encountered fewer stressful life events in the first decade of life and had fewer health problems in childhood and adolescence; girls who were more autonomous as toddlers had fewer health problems in each decade of life and fewer coping problems by age 40 (Werner and Smith, 1992).

### A Refined Model: The Mediation Role of Resilience From Basic Psychological Needs to Performance

Proposed a “Teacher-Parents-Peers” model illustrating the possible pathway influencing resilience of the students from the lens of a motivational model. The model holds that social partners are working as social resources by supporting the needs of the students for relatedness, competence, and autonomy (Deci and Ryan, 1985; Connell and Wellborn, 1991; Skinner, 1995). When the social resources transit to personal resources, that is, when the needs are met, students could construct their learning motivation by promoting positive self-perceptions of relatedness, competence, and autonomy. Students can thus draw on the resources of self-determination when they encounter difficulties, coping constructively, reengaging with challenging academic tasks, and in general developing everyday motivational resilience or academic buoyancy (Martin and Marsh, 2008; Skinner et al., 2008; Skinner and Pitzer, 2012; Aydin and Michou, 2019).

Skinner et al. (2013) showed that all the three components of human basic needs positively correlated to adaptive behaviors, such as strategizing, help-seeking, self-engagement, commitment, etc., and negatively correlated to maladaptive ways, for example, confusion, escape, concealment, projection, etc. Specifically, competence predicted subsequent emotional resilience and coping strategies in school; relatedness was positive to academic coping and reengagement; nevertheless, autonomy had no obtained influences (Pitzer and Skinner, 2017). At the same time, motivational resilience, in turn, predicted improvements in the achievements of students and also provided feedback to increase their personal and interpersonal resources (Skinner et al., 2016).

### A Confucian Culture Perspective

In many international comparison studies, Chinese and other Asian students outperformed students from other cultures (e.g., OECD, 2013, 2016; Mullis et al., 2016). The social-cultural explanation mainly focuses on the Confucianism wisdom on learning, which emphasizes that the survival and development of everyone were in social relations (Li, 2012). Therefore, Chinese students regard learning as a process of self-cultivation at the moral and social levels, unlike western wisdom, which primarily focuses on knowledge understanding, exploring, and developing (Li and Li, 2002; Li and Yamamoto, 2020). Chinese students usually first set up lofty aspirations in the initial stage of learning irrespective of their intrinsic motivation in SDT perspective (Hau and Ho, 2010; Liu et al., 2020). In case the individual lacks initial interest, taking “commitment” or “value” as the starting point and using external incentive means can significantly improve the academic performance of Chinese students and make them form good learning habits (Li and Fischer, 2004).

Typically, the cultural belief on “commitment” and “value” may work as a protective factor for students in adversity. Among the large proportion of the resilience students in Asian countries and organizations, the prominent facilitators are identified motivation and academic aspiration (Sandoval-Hernández and Białowolski, 2016; Avvisati, 2018). This is typically embedded in Confucianism which advocates that there is no religion, and the way of sages is not the privilege of the elite, but an ideal that all learners regard as the ultimate goal. This combination of moral cultivation, academic achievements, political forces, social status, and economic benefits has led to the highest value of learning in Chinese culture (Li, 2012).

## The Present Study

**Figure 1:**
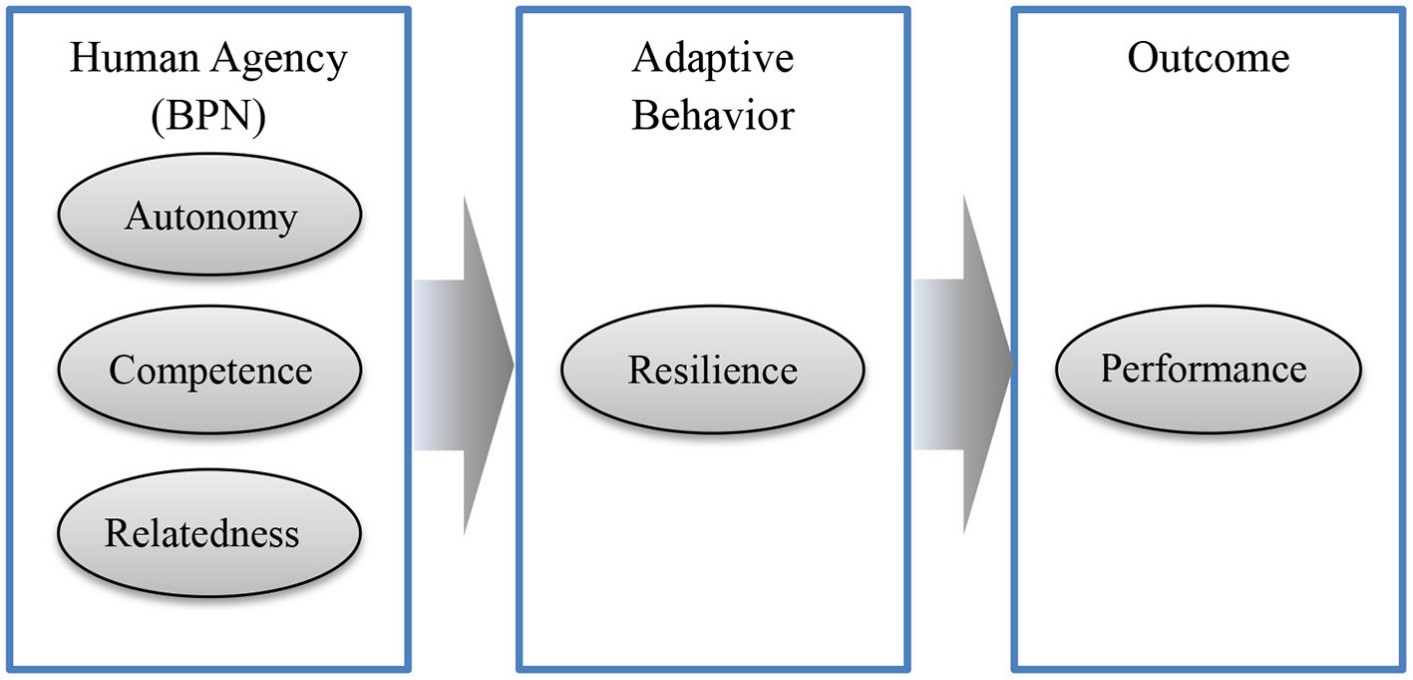
A theoretical framework of the mediation role of resilience from human agency to academic performance. BPN, basic psychological needs.

We can make a further step on the model of Skinner and colleagues and propose that resilience may mediate the process from the human agency to academic outcomes. As claimed from the SDT, the human agency plays a universal, essential, and inherent role, whose needs are like the root trait of any adaptive behavior (Vansteenkiste et al., 2020). This indicates that the role of the basic psychological needs should be primary resources or antecedences of the human adaptive dispositions. Once individuals bounce to a normal state and make effort, it is easy to predict their better academic performance (Figure 1). The first aim of the present study is to test the proposed mediation model. Particularly, we used a longitudinal study to show stronger evidence in this chained mediation model.

Particularly, during adolescents, the sense of self-shift toward more abstract portrayals that began in middle childhood continues. The capacity for mutual understanding and the knowledge that others are unique individuals with feelings of their own also contribute to a dramatic increase in self-disclosure, intimacy, and loyalty among friends, and to their increasing needs for relatedness especially among friends and peers (Rice and Dolgin, 2008). Moreover, in the Chinese context, adolescents have much longer learning time than western learners as congruently showed in the previous literature, for example, the learning time of 15-year-old students in China or Singapore spent in school per week was at least two times that in Iceland, Ireland, or Norway (Watkins, 2003; OECD, 2016, 2021). Nevertheless, excessive learning time makes learning a burden for students (Tang and Fu, 2008; Zhang et al., 2021). The second aim of the study is to focus on a group of Chinese adolescents who is under heavy learning stress and whose learning agency is necessary to be increased positively.

In this study, we proposed that (i) the basic psychological needs, that is, the needs for relatedness, competence, and autonomy could positively predict academic resilience; (ii) academic resilience could enhance academic performance, after controlling for the necessary background information; (iii) resilience could mediate the path from basic psychological needs to academic performance; and (iv) such a mediation model is effective in a Chinese adolescents group.

## Method

### Objectives

The participants are from a western city in mainland China, with 455 students in their Grade 10 Fall semester. A cluster sampling method was used to get the information from a whole grade. The average age of the students is 15.34 years, with a standard deviation of 0.60 years. Among the 455 students, 264 were girls. There were five participants not completing the survey; so finally, the data of 450 students were retrieved. We collected their academic performance, basic psychological needs, and resilience in the middle term of the semester, and their academic performance again at the end of the semester.

### Measures

#### Academic Performance

The academic performance was recorded as the test scores. We tracked two large-scale standardized tests across the grade, namely: the middle term test (in November) and the final term (in January) test. The midterm test was used as the control for the previous academic level. Particularly, we selected the domain of mathematics, which is a compulsory class across majors in the region we selected. The mathematics items in 10th grade is mainly about the concept, characteristics, and applications of functions, for example, the monotonicity of functions, the root of quadratic function, solution of inequality, etc.

#### Basic Psychological Needs

The instrument was from the basic psychological need satisfaction scale (Deci and Ryan, 2000). It had three subdimensions, each representing autonomy, competence, and relatedness. This 21-item scale addressed the satisfaction of the needs, in general, in the life of an individual, and was adapted from a more broadly used measure of need satisfaction at work (Kasser et al., 1992; Ilardi et al., 1993; Deci et al., 2001). The Chinese version of a 24-item scale was introduced and validated by Chen et al. (2015). The original scale did not fit our data well due to some reversed wording problems. Thus, we deleted the items with low loadings and validated the scale by the factor analysis procedure and created a short form of the scale. Finally, we left five items on autonomy, five items on competence, and six items on relatedness. They were Lickert items with “1 = strongly disagree” to “7 = strongly agree” (see Appendix 1 for details). Since there was potential negative wording effect of the scale, a bifactor model with a negative wording effect factor was used to adjust the model fit (Vecchione et al., 2014; Gu et al., 2015). This revised scale had a good model fit [χ^2^ = 280; *df* = 92; *p* < 0.001; CFI = 0.948 (> 0.900); TLI = 0.932 (>0.900); RMSEA = 0.067 (<0.080)]. The adjusted component reliability (Brunner and SÜβ, 2005; Gu et al., 2015) were 0.724, 0.696, and 0.831, respectively for the three subscales. The subscales correlated with each other, and autonomy with competence was 0.949, relatedness with competence was 0.738, and autonomy with relatedness was 0.618. We used a second order factor, i,e., basic psychological need, in the subsequent data analyses.

#### Academic Resilience

The Academic resilience in mathematics scale was used, as it was originally administered to seventh- and eighth-grade students (Ricketts et al., 2017). This is a 9-item single-dimensional scale with “1 = strongly disagree” to “6 = strongly agree.” The item “I plan to graduate from high school” was changed to “I plan to graduate from senior high school” to adapt to the target sample. A larger score represented stronger resilience. It had a reliability coefficient of α =0.844. The average score of the nine items was used as the scale score.

#### Controlling Variables

To balance out the influence of background, sex and midterm examination score were controlled, as many studies suggested (Liu et al., 2020). Sex was a dummy variable where 1 represented boys and 0 represented girls. The middle term test was a standardized test across the sample, which was used as a control for the basic ability level of an individual.

### Analyses

A mediation model was built as shown in Figure 1. We used Mplus 7.11 (Muthén and Muthén, 2012) to conduct the Bayesian Structure Equation Model. The 95% critical intervals were obtained from the Bayesian method.

## Results

The descriptive results are presented in Table 1. It showed that the basic psychological needs had moderate correlations with mathematics resilience and low correlations with mathematics performance. The three basic needs were moderately or highly correlated.

**Table 1:** Correlations and descriptives of the target variables.

		**1**	**2**	**3**	**4**	**5**	**6**	**7**
1	Resilience	1.000						
2	Score Final	0.274^***^	1.000					
3	Sex	0.158^***^	0.045	1.000				
4	Score Mid	0.246^***^	0.672^***^	−0.018	1.000			
5	Autonomy	0.498^***^	0.164^***^	0.089	0.135^**^	1.000		
6	Competence	0.498^***^	0.216^***^	0.114^*^	0.177^***^	0.628^***^	1.000	
7	Relatedness	0.344^***^	0.030	−0.049	0.041	0.335^***^	0.386^***^	1.000
	*M*	4.627	82.613	0.416	91.609	5.003	4.623	5.600
	*SD*	0.817	20.451	0.493	19.769	0.946	0.994	0.884

* *p < 0.05*;

** *p < 0.01*;

*** *p < 0.001*.

**Figure 2:**
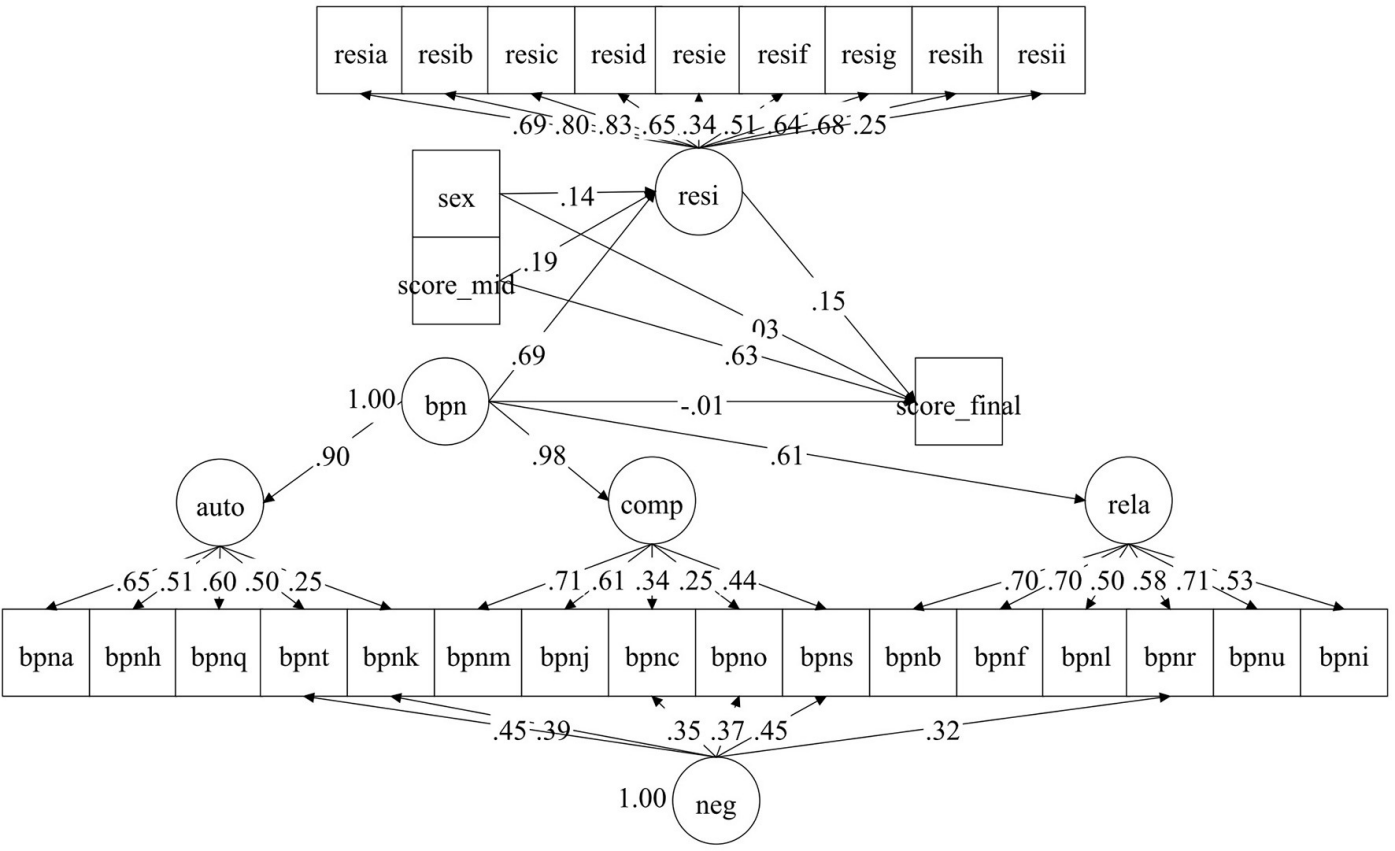
Standardized latent path relationship between basic psychological needs, resilience, and academic performance. Resi, Resilience (items numbered resia to resii); bpn, basic psychological needs (items numbered bpna to bpns); auto, autonomy; rela, relatedness; comp, competence.

The mediation analyses are presented in Figure 2 and Table 2. It showed that after controlling for midterm performance and gender of the students, basic psychological needs improved mathematics resilience (Est. = 0.692, *p* < 0.001).

**Table 2:** Parameter estimates of the relationship between basic psychological needs, resilience, and academic performance (standardized solutions).

	**Est**.	**Posterior S.D**.	***p***	**95% CI**
**Measurement model**
RESI	BY			
RESIA	0.692	0.027	<0.001	0.639, 0.740
RESIB	0.796	0.021	<0.001	0.751, 0.834
RESIC	0.830	0.019	<0.001	0.790, 0.864
RESID	0.649	0.030	<0.001	0.586, 0.702
RESIE	0.344	0.043	<0.001	0.258, 0.426
RESIF	0.511	0.037	<0.001	0.432, 0.580
RESIG	0.637	0.030	<0.001	0.573, 0.691
RESIH	0.680	0.029	<0.001	0.618, 0.732
RESII	0.250	0.046	<0.001	0.159, 0.340
AUTO	BY			
BPNA	0.653	0.037	<0.001	0.575, 0.721
BPNH	0.505	0.044	<0.001	0.415, 0.586
BPNQ	0.600	0.038	<0.001	0.522, 0.672
BPNT	0.502	0.041	<0.001	0.419, 0.579
BPNK	0.252	0.051	<0.001	0.151, 0.352
COMP	BY			
BPNC	0.342	0.048	<0.001	0.238, 0.427
BPNO	0.247	0.050	<0.001	0.148, 0.342
BPNJ	0.608	0.036	<0.001	0.532, 0.676
BPNM	0.709	0.032	<0.001	0.640, 0.767
BPNS	0.438	0.044	<0.001	0.347, 0.521
RELA	BY			
BPNB	0.701	0.031	<0.001	0.637, 0.757
BPNF	0.696	0.032	<0.001	0.630, 0.753
BPNL	0.499	0.041	<0.001	0.416, 0.579
BPNR	0.576	0.037	<0.001	0.499, 0.644
BPNU	0.708	0.033	<0.001	0.638, 0.765
BPNI	0.525	0.040	<0.001	0.443, 0.601
NEG	BY			
BPNT	0.450	0.055	<0.001	0.340, 0.555
BPNK	0.392	0.064	<0.001	0.261, 0.514
BPNC	0.348	0.060	<0.001	0.219, 0.457
BPNO	0.367	0.060	<0.001	0.245, 0.477
BPNS	0.445	0.059	<0.001	0.328, 0.556
BPNR	0.315	0.058	<0.001	0.202, 0.426
BPN	BY			
AUTO	0.896	0.030	<0.001	0.833, 0.942
COMP	0.985	0.008	<0.001	0.966, 0.994
RELA	0.606	0.044	<0.001	0.517, 0.687
**Structural Model**				
RESI	ON			
BPN	0.692	0.037	<0.001	0.617, 0.761
RESI	ON			
SEX	0.144	0.040	<0.001	0.064, 0.222
SCORE_MID	0.186	0.041	<0.001	0.098, 0.261
SCORE_FINA	ON			
BPN	−0.008	0.068	0.458	−0.138, 0.124
RESI	0.146	0.065	0.014	0.014, 0.273
SCORE_FINA	ON			
SCORE_MID	0.632	0.028	<0.001	0.575, 0.685
SEX	0.029	0.035	0.215	−0.040, 0.097

*RESI, Resilience (items numbered RESIA to RESII); BPN, basic psychological needs (items numbered BPNA to BPNS); AUTO, autonomy; RELA, relatedness; COMP, competence; NEG, negative wording effect; SCORE_MID, midterm test score; SCORE_FINA, final est score*.

Resilience could facilitate mathematics performance after controlling for the previous academic level. It had a moderate positive influence which meant that stronger resilience led to higher subsequent performance. It is also noted that basic psychological needs no longer had direct influences on mathematics performance. The standardized indirect effect was 0.100, with a 95% CI of [0.010, 0.192] and a *p* value of 0.014. This meant that resilience mediated the relationship between basic psychological needs and mathematics performance.

## Discussion

The present study generally supports the theoretical model, that the mediation effect of mathematics resilience is supported as influenced by the basic psychological needs and influence to mathematics performance. As SDT suggests, basic psychological needs are universal, inherent, and essential to human agency, and thus would produce adaptive behavior and facilitate individuals to achieve growth (Vansteenkiste et al., 2020). When the needs are satisfied, individuals are likely to feel autonomic, self-determined, and intrinsically motivated. Subsequently, individuals work and take efforts to come over the difficulties and fulfill it themselves, which would be shown as everyday resilience in congruence with the previous research (e.g., Skinner et al., 2016). Particularly, the present study proved such a relationship in mathematics, where the domain-specific resilience is rather close to the relevant performance (Collie et al., 2016).

We have shown that the mediation model is particularly effective in the Chinese adolescent group. One possible reason is that eastern learners hold a more positive attitude and respect diligence more than western learners, influenced by the daily communication with their partners, peers, and parents (Li, 2012; Fu and Markus, 2014; Li and Yamamoto, 2020). This positive attitude toward diligent studying may encourage eastern learners to motivate conceptual and meaningful learning from the repeated revisions and the in-school examinations, which are more frequently administrated in senior high schools in Asian countries (Zhang et al., 2021). The positive attitude toward examinations provides more informational feedback through the learning process of the students and strengthens their self-reference, with competence being the highest weight among the three basic psychological needs.

Although the mediation role of resilience is generally supported, the direct influence from satisfying basic needs to academic performance is not salient in the mediation model. One possibility may be the domain difference between the BPNT and the performance since the BPNT was on the general human agency whereas the outcome focused on the mathematics domain. Another alternative is that despite the controlling for the previous test scores, the “change” of the test score may not be influenced during such a short time interval (i.e., the interval between midterm and final examinations was only 3 months, which may hardly lead to substantial changes in academic performance). The change of the test score could be influenced by many factors such as the previous knowledge, cognition/metacognition, and testing exertion; nevertheless, it could be promoted by satisfying basic psychological needs not that directly (Ryan and Deci, 2017). The change of test score is thus a lagged outcome when originating to the psychological satisfaction, where subjective well-being, adaptation, intrinsic motivation, etc. may mediate such a pathway (Skinner et al., 2013, 2016). In the present study, the mediation model successfully supports the close relationship between basic psychological needs and the resilience disposition, but when it comes to a long way to the destination test scores, more evidence or longer time interval is needed.

## Limitations and Future Directions

In the present study, the quality of the basic psychological needs scale should be noticed. There were wording effects of the negative items and the correlation of the three subscales with each other was so high. To eliminate measurement error, the bifactor model was used to reduce the negative wording effect, and alternative reliability estimates were more suitable after this adjustment (Gu et al., 2015; McNeish, 2018). This adjustment is necessary for the subsequent analysis since the component reliability was thus considerable (approximately larger than 0.70). As for the highly related subconstruct, we ran an alternative one-factor model which showed an α coefficient of 0.814, indicating that the three basic needs could be integrated. So we used a second-order CFA to finally model the basic psychological needs construct, which shows that these needs may inherently be highly correlated with each other. The BPNT scale needs further information about its structure and implications.

The assumption is that the basic needs as the antecedences of the dynamic process mainly comes from the SDT perspective, as guided in the present study; however, our empirical results due to the simultaneous collection of the data could not reflect such a causal relationship whether the human agency is indeed the “origin” of resilience. It is possible that if individuals are disposed toward high resilience, they are likely to change and adjust their feeling or knowledge of needs satisfaction (Waterschoot et al., 2019). In the future study, the construct of resilience, as well as the basic psychological needs may also be designed in subsequent data collection. As a result, a potential “reciprocal relationship” between the basic psychological needs and resilience may be examined.

## Conclusion

The psychological basic needs could strengthen academic resilience. Autonomy, competence, and relatedness may simultaneously enhancing the feeling of resilience. Meanwhile, academic resilience shows its mediating role between basic psychological needs and academic performance; satisfaction of basic psychological needs would facilitate subsequent performance through the positive role of resilience.

## Data Availability Statement

The raw data supporting the conclusions of this article will be made available by the authors, without undue reservation.

## Ethics Statement

The studies involving human participants were reviewed and approved by Faculty of Psychology, Southwest University. Written informed consent to participate in this study was provided by the participants' legal guardian/next of kin.

## Author Contributions

YL designs the research, analyzes the data, and writes the article. XH conducts the research and analyzes the data. All authors contributed to the article and approved the submitted version.

## Conflict of Interest

The authors declare that the research was conducted in the absence of any commercial or financial relationships that could be construed as a potential conflict of interest.

## Publisher's Note

All claims expressed in this article are solely those of the authors and do not necessarily represent those of their affiliated organizations, or those of the publisher, the editors and the reviewers. Any product that may be evaluated in this article, or claim that may be made by its manufacturer, is not guaranteed or endorsed by the publisher.
