# Supplementary material for: Effects of Basic Psychological Needs on Resilience: A Human Agency Model
Source: Front Psychol. 2021 Aug 31;12:700035. doi: 10.3389/fpsyg.2021.700035 (PMC8438124; doi:10.3389/fpsyg.2021.700035)
Supplement: Supplementary file 1 [file Table_1.DOCX]

Appendix 1. Factor Loadings of Basic Psychological Needs Scale

|  | Est. | S.E. | Est./S.E. |
| --- | --- | --- | --- |
| Autonomy |  |  |  |
| I feel like I am free to decide for myself how to live my life. | 0.624 | 0.032 | 19.528*** |
| I generally feel free to express my ideas and opinions. | 0.602 | 0.035 | 17.077*** |
| I feel like I can pretty much be myself in my daily situations. | 0.593 | 0.036 | 16.589*** |
| There is not much opportunity for me to decide for myself how to do things in my daily. (R) | 0.799 | 0.063 | 12.642*** |
| In my daily life, I frequently have to do what I am told. (R) | 0.487 | 0.075 | 6.531*** |
|  |  |  |  |
| Competence |  |  |  |
| Often, I do not feel very competent. (R) | 0.579 | 0.085 | 6.806*** |
| In my life I do not get much of a chance to show how capable I am. (R) | 0.523 | 0.086 | 6.054*** |
| I have been able to learn interesting new skills recently. | 0.683 | 0.034 | 20.305*** |
| Most days I feel a sense of accomplishment from what I do. | 0.673 | 0.029 | 22.893*** |
| I often do not feel very capable. (R) | 0.701 | 0.073 | 9.570*** |
|  |  |  |  |
| Relatedness |  |  |  |
| I really like the people I interact with. | 0.697 | 0.028 | 24.595*** |
| I get along with people I come into contact with. | 0.777 | 0.029 | 26.684*** |
| People in my life care about me. | 0.557 | 0.035 | 15.752*** |
| The people I interact with regularly do not seem to like me much. (R) | 0.982 | 0.152 | 6.457*** |
| People are generally pretty friendly towards me. | 0.778 | 0.031 | 25.063*** |
| I consider the people I regularly interact with to be my friends. | 0.508 | 0.037 | 13.581*** |

*Note*. *** *p* < .001

R = Reversed/Negative Wording Items
